# Chronic Exposure to Nicotine Enhances Insulin Sensitivity through α7 Nicotinic Acetylcholine Receptor-STAT3 Pathway

**DOI:** 10.1371/journal.pone.0051217

**Published:** 2012-12-12

**Authors:** Tian-Ying Xu, Ling-Ling Guo, Pei Wang, Jie Song, Ying-Ying Le, Benoit Viollet, Chao-Yu Miao

**Affiliations:** 1 Department of Pharmacology, Second Military Medical University, Shanghai, China; 2 Key Laboratory of Nutrition and Metabolism, Institute for Nutritional Sciences, Shanghai Institutes for Biological Sciences, Chinese Academy of Sciences, Shanghai, China; 3 Institut Cochin, Université Paris Descartes, CNRS (UMR8104), Paris, France; 4 Inserm, U567, Paris, France; H. Lee Moffitt Cancer Center & Research Institute, United States of America

## Abstract

This study was to investigate the effect of nicotine on insulin sensitivity and explore the underlying mechanisms. Treatment of Sprague-Dawley rats with nicotine (3 mg/kg/day) for 6 weeks reduced 43% body weight gain and 65% blood insulin level, but had no effect on blood glucose level. Both insulin tolerance test and glucose tolerance test demonstrated that nicotine treatment enhanced insulin sensitivity. Pretreatment of rats with hexamethonium (20 mg/kg/day) to antagonize peripheral nicotinic receptors except for α7 nicotinic acetylcholine receptor (α7-nAChR) had no effect on the insulin sensitizing effect of nicotine. However, the insulin sensitizing effect but not the bodyweight reducing effect of nicotine was abrogated in α7-nAChR knockout mice. Further, chronic treatment with PNU-282987 (0.53 mg/kg/day), a selective α7-nAChR agonist, significantly enhanced insulin sensitivity without apparently modifying bodyweight not only in normal mice but also in AMP-activated kinase-α2 knockout mice, an animal model of insulin resistance with no sign of inflammation. Moreover, PNU-282987 treatment enhanced phosphorylation of signal transducer and activator of transcription 3 (STAT3) in skeletal muscle, adipose tissue and liver in normal mice. PNU-282987 treatment also increased glucose uptake by 25% in C2C12 myotubes and this effect was total abrogated by STAT3 inhibitor, S3I-201. All together, these findings demonstrated that nicotine enhanced insulin sensitivity in animals with or without insulin resistance, at least in part via stimulating α7-nAChR-STAT3 pathway independent of inflammation. Our results contribute not only to the understanding of the pharmacological effects of nicotine, but also to the identifying of new therapeutic targets against insulin resistance.

## Introduction

Insulin resistance occurs in 20%–25% of the human population [1]. It is a chief component of type 2 diabetes mellitus and an important risk factor for cardiovascular disease as well as certain forms of cancer [2]–[5]. Since the commonly used insulin sensitizer thiazolidinediones, selective agonists for nuclear peroxisomal proliferator-activated receptor-γ, have been reported to be associated with increased risk of massive hepatic necrosis, heart failure, and bladder cancer in patients treated with these drugs [6]–[8], it is of great value to identify new therapeutic targets for development of novel therapy against insulin resistance.

Smoking cigarette has been associated with insulin resistance [9]. As a major constituent of tobacco, nicotine has long been considered to induce insulin resistance, but till now, results from clinical and animal studies are contradictory. Clinical studies reported that nicotine infusion acutely impairs insulin sensitivity in type 2 diabetic patients and smokers but not in healthy subjects [10], [11]. Long-term nicotine gum or nicotine patch replacement in previous smokers is associated with insulin resistance [12], [13]. However, animal studies show that long-term oral nicotine administration reduces insulin resistance in obese rats [14]. In fact, clinical studies may have more influencing factors. Smoking history may complex the outcome of nicotine in examined subjects because approximately 4000 compounds exist in cigarette smoke. Recent in vitro study suggests that nicotine may have opposite action on insulin sensitivity when treating temporarily or chronically [15]. Thus, more evidence for the effect of nicotine on insulin sensitivity is needed to be provided on different animal models and the underlying mechanism is needed to be clarified.

In our previous study, we were surprised to find that chronic nicotine treatment can significantly reduce HOMA of insulin resistance (HOMA-IR) in normal rats, suggesting that nicotine may enhance insulin sensitivity [16]. In the present study, to further study this phenomenon, we treated normal rats with nicotine for 6 weeks and examined insulin sensitivity by detecting blood glucose and insulin levels, and performing insulin tolerance test and glucose tolerance test. Nicotinic acetylcholine receptor (nAChR) antagonist, α7-nAChR agonist, signal transducer and activator of transcription 3 (STAT3) inhibitor, α7-nAChR knockout (α7-nAChR^−/−^) and AMP-activated kinase-α2 knockout (AMPKα2^−/−^) mice were used to indentify the nAChR subtypes mediating the effect of nicotine on insulin sensitivity and explore the underlying mechanisms. We demonstrated that chronic treatment of nicotine enhanced insulin sensitivity in normal rodents through α7-nAChR-STAT3 pathway which is independent of the anti-inflammatory effect of nicotine. Activation of α7-nAChR also improved insulin sensitivity in AMPKα2^−/−^ mice, a model of insulin resistance.

## Materials and Methods

### Ethics Statement

All animals received human care and all study protocols were approved by the Institutional Animal Care and Use Committee of Second Military Medical University, China.

### Chemicals

Nicotine was purchased from U-sea Biotech Co., Ltd., Shanghai, China. PNU-282987 and hexamethonium chloride were purchased from Sigma (St. Louis, MO). 2-(N-(7-nitrobenz-2-oxa-1,3-diazol-4-yl)amino)-2-deoxyglucose (2-NBDG) was from Invitrogen. STAT3 specific inhibitor, S3I-201, was provided by Santa Cruz Biotechnology (Santa Cruz, CA).

### Animals and genotyping

Male Sprague-Dawley rats were purchased from Sino-British SIPPR/BK Lab Animal Ltd, Shanghai, China. α7-nAChR^−/−^ mice, AMPKα2^−/−^ mice were generated and genotyped by PCR analysis as described previously [17]–[19].

### Animal treatment

To study the effect of nicotine on insulin sensitivity, Sprague-Dawley rats aged 10–11 weeks were divided into two groups. The control group received subcutaneous injection of saline; the nicotine group received subcutaneous injection of nicotine (3 mg/kg/day). Body weight was measured once a week. Insulin tolerance test, glucose tolerance test were performed and blood samples were collected for biochemical assays after 6 weeks of treatment.

To identify the nAChR subtypes involved in nicotine-induced increase of insulin sensitivity, Sprague-Dawley rats were treated with saline or hexamethonium (20 mg/kg, i.p.) 20 minutes before subcutaneous injection of saline or nicotine (3 mg/kg) every day. Male α7-nAChR^−/−^ mice aged 8–9 weeks were subcutaneously injected with saline or nicotine (3 mg/kg/day). Body weight was measured once a week. After treatment for 6 weeks, blood samples were collected for biochemical assays.

To examine whether selective α7-nAChR agonist, PNU-282987, can enhance insulin sensitivity, male C57BL/6J mice aged 8–9 weeks were subcutaneously injected with saline or PNU-282987 (0.53 mg/kg/day) [20], [21]. Body weight was measured once a week. Insulin tolerance test, glucose tolerance test were performed after 6 weeks of treatment.

To find out downstream molecules of α7-nAChR to enhance insulin sensitivity, male AMPKα2^−/−^ mice and C57BL/6J mice aged 8–9 weeks were subcutaneously injected with saline or PNU-282987 (0.53 mg/kg/day) for 6 weeks. In AMPKα2^−/−^ mice, body weight was examined once a week. Insulin tolerance test and glucose tolerance test were performed and blood samples were collected for biochemical assays at the end of treatment. In C57BL/6J mice, the phosphorylation of STAT3 in skeletal muscle, visceral adipose and liver were examined by Western blot after 6 weeks of treatment.

### Insulin tolerance test (ITT)

Rats were injected with insulin (0.25 IU/kg, i.p.) after an over-night fast, while mice were injected with insulin (0.55 IU/kg, i.p.) after 6-hour fast. Blood glucose levels were measured at indicated times with a portable glucose meter (LifeScan, Milpitas, CA) after tail snipping [19], [22]–[24].

### Glucose tolerance test (GTT)

Glucose (2.0 g/kg) was given to mice (i.p.) and rats (i.g.) after an over-night fast. Blood glucose levels were then measured at indicated times with a portable glucose meter (LifeScan, Milpitas, CA) after tail snipping. Simultaneously, blood samples were collected for examining insulin concentration [19], [23], [25].

### Cell Culture and differentiation

Mouse C2C12 myoblasts were purchased from American Type Culture Collection and cultured with Dulbecco's modified Eagle's medium supplemented with 10% (v/v) FBS, 2 mmol/L glutamate, 15 mmol/L HEPES, 500 IU/mL penicillin, and 100 mg/mL streptomycin in 95% O_2_ and 5% CO_2_. To obtain fully differentiated myotubes, FBS was removed from cell culture at 70% confluence. Cells were incubated in a medium containing only 2% (v/v) horse serum for 4 additional days [19], [26].

### Glucose Uptake Assay

Glucose uptake was measured using a nonradioactive fluorescent glucose 2-NBDG method, as described previously [19], [27]. Briefly, cells were either left untreated or preincubated with one of the following treatments for 48 hours prior to treatment with 100 nM insulin for 15 min: 30 µM PNU-282987; 30 µM PNU-282987 plus 100 µM S3I-201. Subsequently, 100 µM 2-NBDG solution was added and the cells with 2-NBDG were incubated for an additional 10 min. The fluorescence retained in the cells was measured with a microplate fluorimeter (Infinite M200; Tecan, Hillsborough, NC), set at an excitation wavelength of 488 nm and an emission wavelength of 542 nm [28].

### Immunoblotting

Phosphorylation of STAT3 was examined by SDS-PAGE and immunoblotted as described previously [18], [29]. Antibodies against phosphorylated or total STAT3 were from Cell Signaling Technology (MA, USA). Antibodies against α7-nAChR were from Millipore (MA, USA). Secondary antibodies were IRDye800CW goat anti-rabbit IgG (LI-COR Biosciences, Nebraska, USA). The images were captured and analyzed by the Odyssey infrared fluorescence imaging system (Li-Cor Bioscience) [30]. Each experiment was repeated at least three times.

### Blood biochemical assays

Blood glucose and triglyceride levels were measured with an autoanalyzer (Beckman Autoanalyzer; Beckman Instruments, Fullerton, CA, USA). Rat insulin levels were measured by radioimmunoassay with a kit from Tianjin Jiuding Medical Bioengineering (Tianjin, China). This assay has a limit of detection of 0.92 µIU/mL, with interassay and intraassay coefficients of variation of 7.6% and 12.2%, respectively. Mice insulin levels were determined by enzyme-linked immunosorbent assay (ELISA) according to the manufacturer's instructions (Mercodia, Uppsala, Sweden). This assay has a limit of detection of ≤0.10 µg/L, with interassay and intraassay coefficients of variation of 2.3% and 5.1%, respectively. The homeostasis model assessment of insulin resistance (HOMA-IR) index was calculated using the following formula [31], [32]: HOMA-IR = fasting blood insulin (mIU/L)×fasting blood glucose (mmol/L)/22.5. The quantitative insulin sensitivity check index (QUICKI) was calculated according to the original formula [33] as the inverse log sum of fasting insulin in mIU/L and fasting glucose in mg/dl. QUICKI = 1/[log(fasting blood glucose)+log(fasting blood insulin)].

### Statistical analysis

Data are expressed as the mean ± SEM. Statistical comparisons between two groups were performed by Student's *t* test. Comparisons among several groups (≥3 groups) were performed by analysis of variance followed by Tukey's post hoc test. Statistical significance was set at P<0.05.

## Results

### Chronic nicotine treatment enhances insulin sensitivity in normal rats

**Figure 1:**
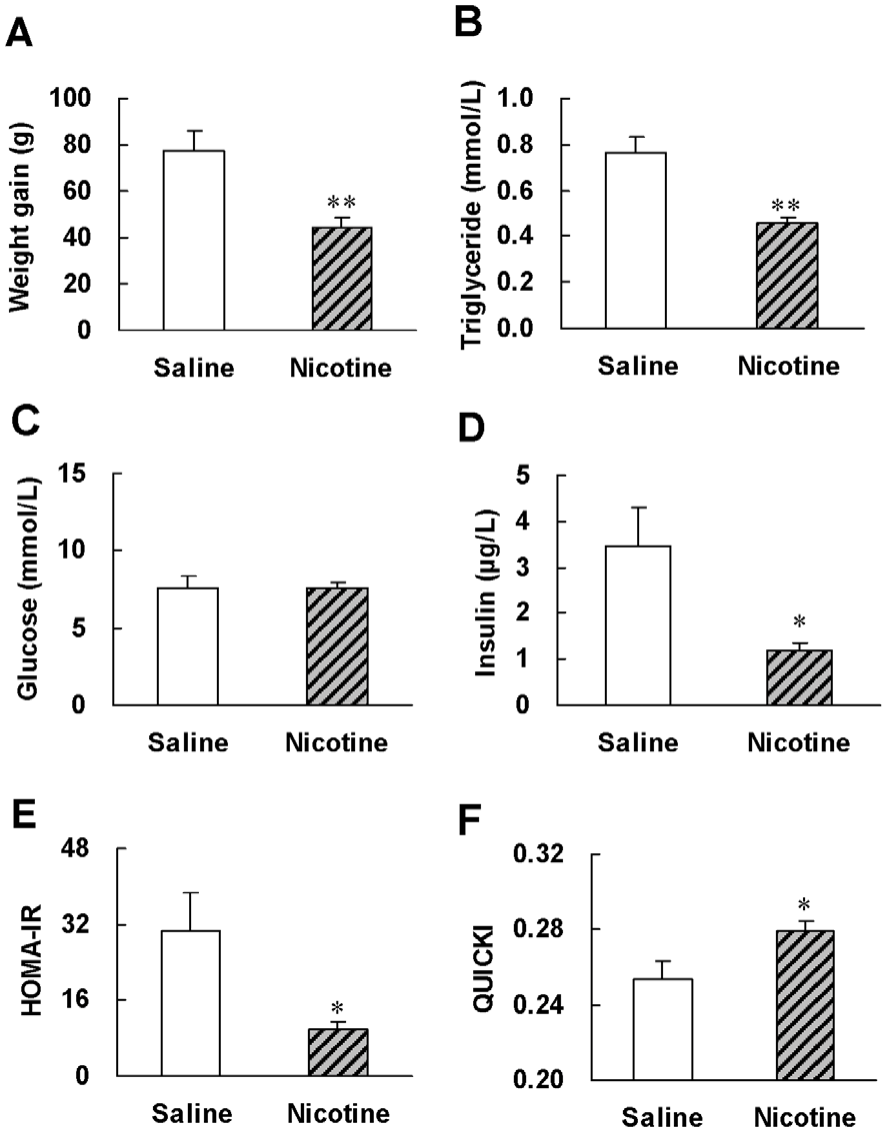
Chronic nicotine treatment reduces body weight and improves basal metabolic parameters as well as insulin sensitivity indexes in normal rats. After 6 weeks of saline or nicotine treatment, (A) weight gain, (B, C, D) basal metabolic parameters and (E, F) insulin sensitivity indexes were evaluated. Data are means ± SE (n = 6–8). ^*^ *P*<0.05, ^**^ *P*<0.01 *vs* saline treatment.

After 6 weeks of treatment, the weight gain in nicotine-treated rats was only 57% of that in saline-treated rats (Fig. 1A). Blood triglyceride levels showed a 40% reduction after nicotine treatment (Fig. 1B). Nicotine treatment significantly reduced 65% basal insulin level (Fig. 1D) but had no effect on glucose level (Fig. 1C), indicating a higher insulin sensitivity in nicotine treated rats. Nicotine treated-rats reduced HOMA-IR to 35% and elevated QUICKI indexes to 112% of that in saline-treated rats (Fig. 1, E and F), supporting that chronic nicotine treatment enhances insulin sensitivity.

**Figure 2:**
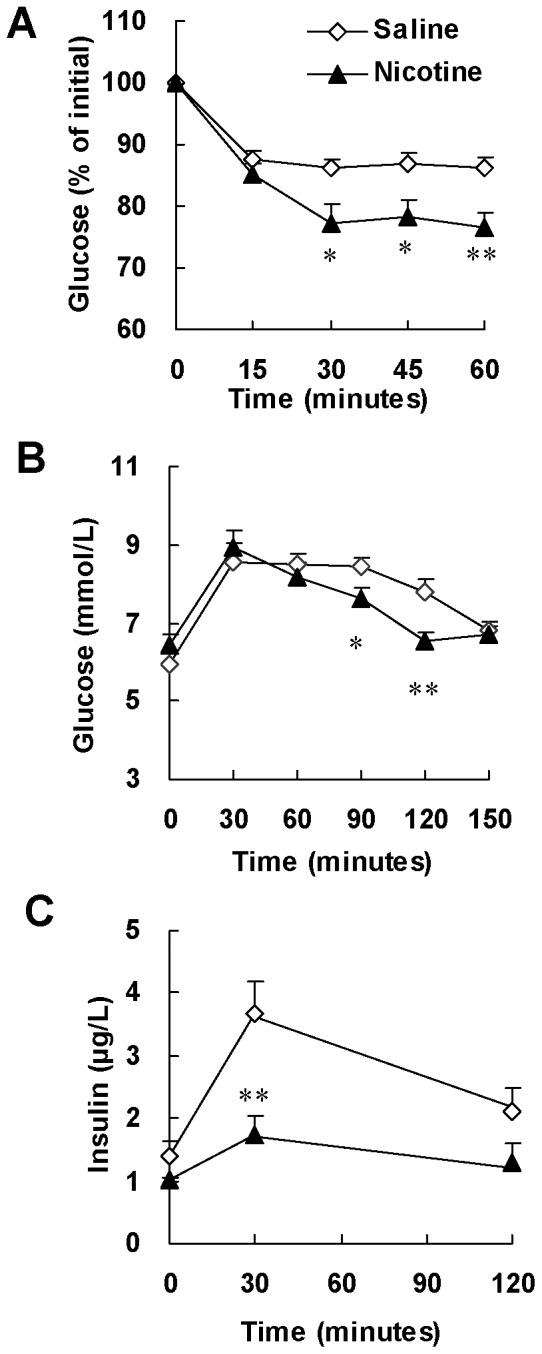
Chronic nicotine treatment enhances insulin sensitivity in normal rats. After 6 weeks of saline or nicotine treatment, (A) insulin tolerance test (ITT) and (B, C) glucose tolerance test (GTT) were performed. ITT was performed in overnight fasted rats with insulin challenge at 0.25 IU/kg of body weight (i.p.). GTT was performed in overnight fasted rats with glucose challenge at 2.0 g/kg of body weight (i.g.). Data are means ± SE (n = 7). ^*^ *P*<0.05, ^**^ *P*<0.01 *vs* saline treated rats.

We further performed ITT and GTT to evaluate insulin sensitivity. ITT showed significant decrease of blood glucose levels at 30, 45 and 60 minutes after insulin injection in nicotine-treated rats compared with those in saline-treated rats (Fig. 2A), suggesting that nicotine treatment enhances insulin sensitivity. Meanwhile, GTT showed a more rapid glucose clearance (Fig. 2B) but lower insulin levels (Fig. 2C) in nicotine-treated rats compared with saline-treated animals. Thus, results from both ITT and GTT confirmed that chronic nicotine treatment enhanced insulin sensitivity in normal rats.

### Nicotine enhances insulin sensitivity through activating α7-nAChR

**Figure 3:**
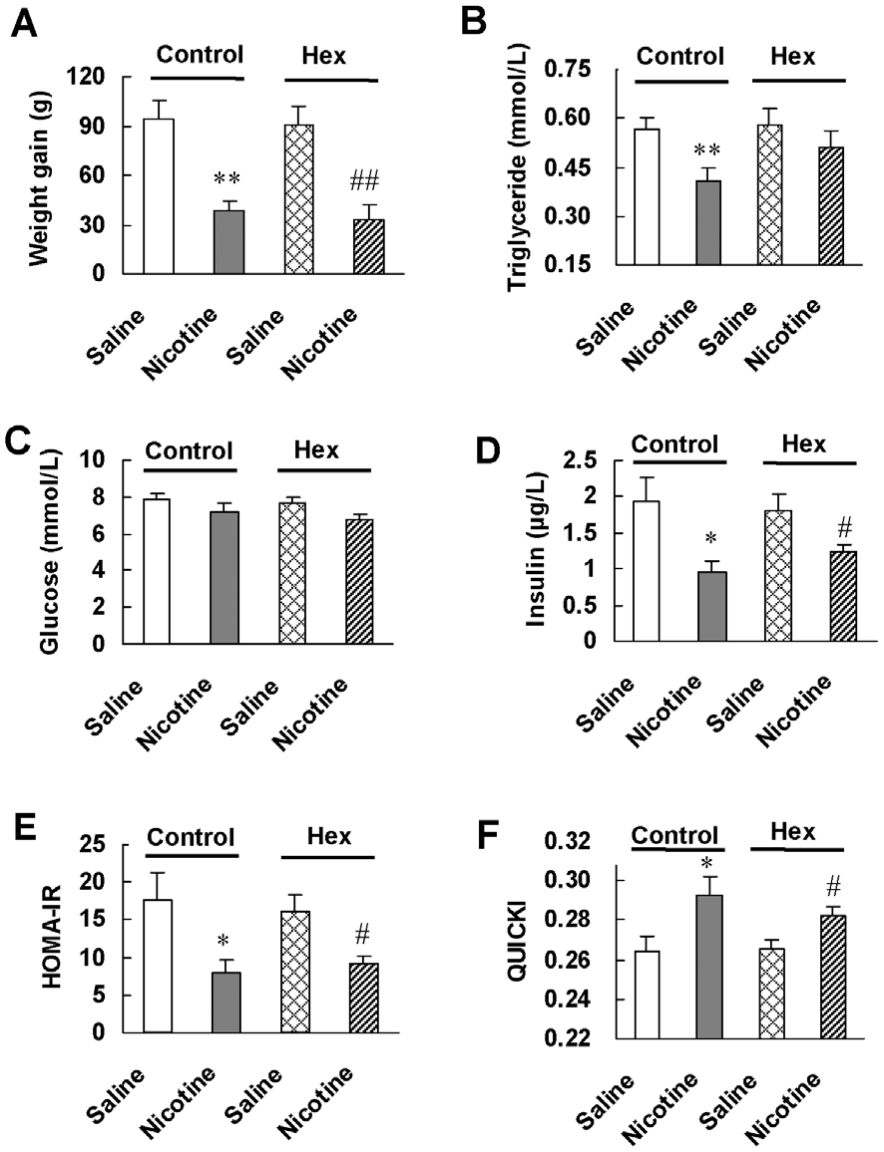
Blocking peripheral nAChRs except for α7-nAChR by hexamethonium pretreatment has no effect on insulin sensitizing effect of nicotine in normal rats. After 6 weeks of treatment with saline followed by saline or nicotine (Control), or with hexamethonium followed by saline or nicotine (Hex), (A) weight gain, (B, C, D) basal metabolic parameters and (E, F) insulin sensitivity indexes were examined. Data are means ± SE (n = 7–8). ^*^ *P*<0.05, ^**^ *P*<0.01 vs Control-Saline; ^#^ *P*<0.05, ^##^ *P*<0.01 *vs* Hex-Saline.

We then studied which nicotinic receptor subtype mediated the insulin sensitizing effect of nicotine. Pretreatment of rats with hexamethonium had no significant effect on nicotine-induced alteration of body weight (Fig. 3A), blood glucose and insulin levels (Fig. 3, C and D), HOMA-IR (Fig. 3E) and QUICKI (Fig. 3F), but significantly reversed nicotine-induced reduction of blood triglyceride level (Fig. 3B). As hexamethonium is a peripheral non-selective antagonist for nAChRs with low potency at α7-nAChR, and a relative low dose of hexamethonium which antagonizes most of the major types of nAChRs except for α7-nAChR [34]–[36] was used in our experiments, these results suggest the involvement of α7-nAChR in the elevation of insulin sensitivity by nicotine.

**Figure 4:**
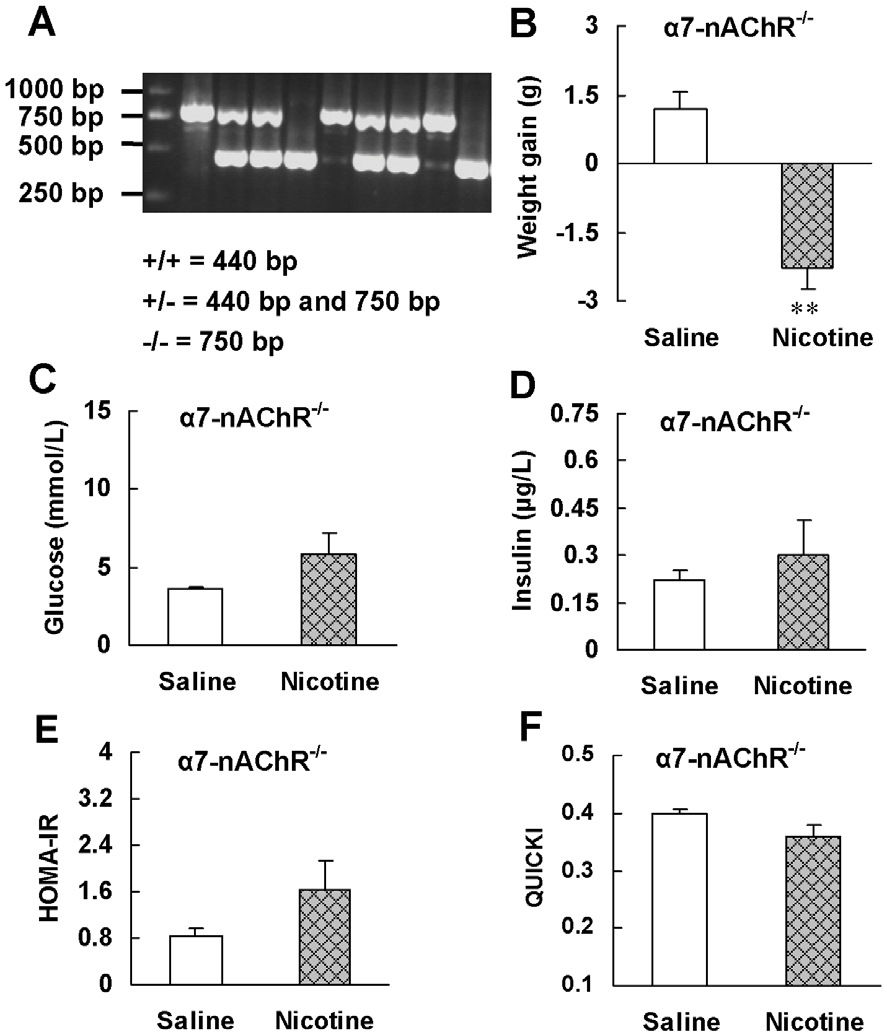
α7-nAChR knockout abrogates insulin sensitizing effect of nicotine. (A) Genotype characterization by PCR analysis of tail DNA from offspring derived from heterozygous intercrosses. Expected fragment sizes of the wild-type mice (+/+; 440 bp), heterozygous knockout mice (+/−, 440 bp and 750 bp) and homozygotous knockout mice (−/−; 750 bp) are shown. Only α7-nAChR homozygotous knockout mice were used for chronic treatment. After 6 weeks of saline or nicotine treatment, (B) weight gain, (C, D) basal metabolic parameters and (E, F) insulin sensitivity indexes were evaluated in α7-nAChR^−/−^ mice. Data are means ± SE (n = 5–6). ^**^ *P*<0.01 *vs* saline treatment.

We further confirmed the involvement of α7-nAChR in the increase of insulin sensitivity by nicotine using α7-nAChR^−/−^ mice. Genotype of wild-type, heterozygous and homozygous α7-nAChR knockout mice was determined by PCR analysis with tail DNA. A representative PCR result is shown in Fig. 4A. Wild-type (+/+) mice gave a 440 bp band, whereas heterozygous (+/−) mice gave an additional 750 bp band and the homozygous (−/−) mice, only a 750 bp band.

After nicotine treatment for 6 weeks, α7-nAChR^−/−^ mice reduced 2.3±0.44 g, 9.2% of initial bodyweight (Fig. 4B). The blood glucose levels in nicotine-treated mice were within normal range and had a tendency to increase compared with those in saline-treated mice (Fig. 4C). α7-nAChR knockout reversed the reduction of blood insulin level and HOMA-IR index as well as the increase of QUICKI index induced by nicotine (Fig. 4, D–F). These results further support that nicotine enhances insulin sensitivity through activation of α7-nAChR.

### Selective activating α7-nAChR can enhance insulin sensitivity

**Figure 5:**
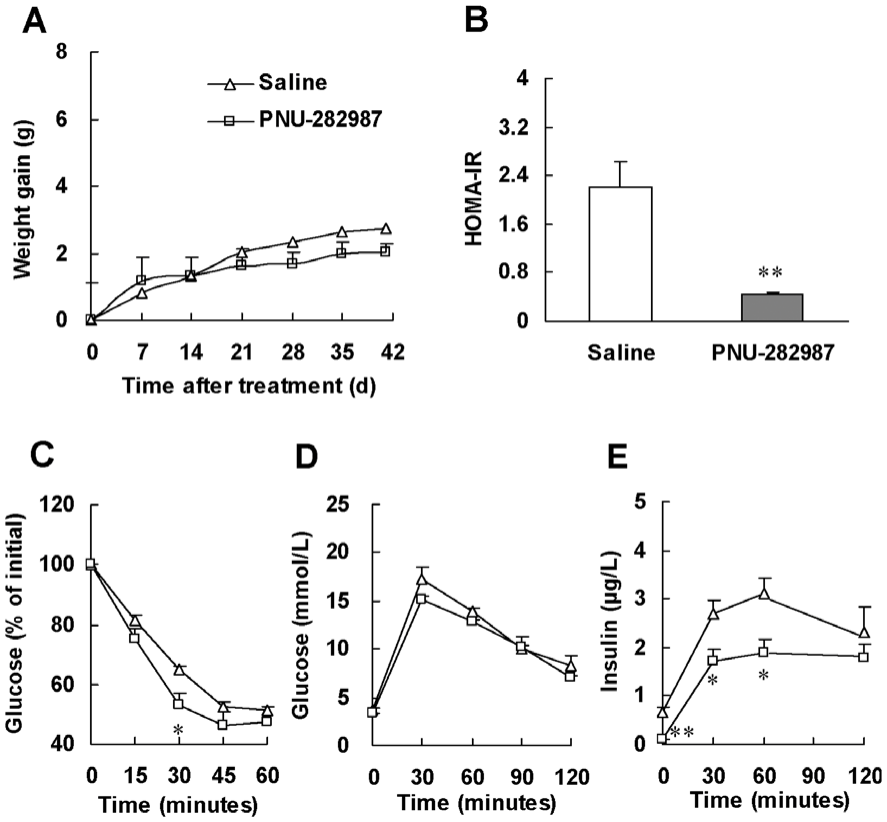
Selective α7-nAChR agonist, PNU-282987, enhances insulin sensitivity in normal mice. (A) Weight gain in mice during PNU-282987 treatment. At the end of PNU-282987 treatment, (B) HOMA-IR, (C) insulin tolerance test (ITT) and (D, E) glucose tolerance test (GTT) were performed. ITT was performed in mice 6 h after food removal with insulin challenge at 0.55 IU/kg of body weight (i.p.). GTT was performed in overnight fasted mice with glucose challenge at 2.0 g/kg of body weight (i.p.). Data are means ± SE (n = 5–6). ^*^ *P*<0.05, ^**^ *P*<0.01 *vs* saline treatment.

To examine whether selective activating α7-nAChR can enhance insulin sensitivity, we treated mice with PNU-282987, a selective agonist for α7-nAChR, for 6 weeks. Compared with treatment with saline, PNU-282987 treatment resulted in similar weight gain but 80% HOMA-IR reduction (*P*<0.01, Fig. 5, A and B). ITT showed 53% significant decrease of blood glucose levels at 30 minute after insulin injection in PNU-282987-treated mice (Fig. 5C). Meanwhile, GTT showed lower blood insulin levels (Fig. 5E) but similar glucose change in PNU-282987-treated mice compared with saline-treated animals. These results demonstrate selective activating α7-nAChR can enhance insulin sensitivity, further confirming the critical role of α7-nAChR in the enhancement of insulin sensitivity by nicotine.

### STAT3 but not AMPKα2 participates in the downstream signal pathway of α7-nAChR to enhance insulin sensitivity

**Figure 6:**
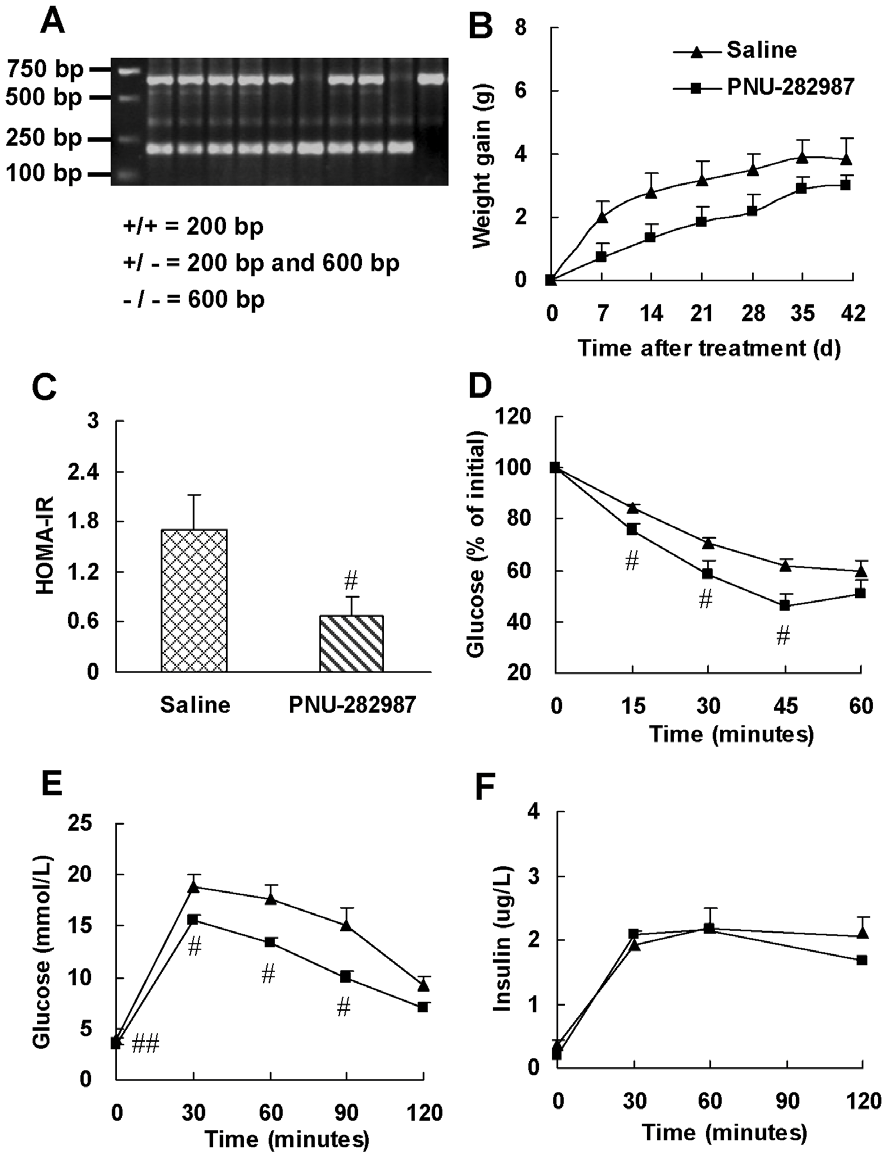
Selective α7-nAChR agonist, PNU-282987, enhances insulin sensitivity in AMPKα2^−/−^ mice. (A) Genotype characterization by PCR analysis of tail DNA from offspring derived from heterozygous intercrosses. Expected fragment sizes of the wild-type mice (+/+; 200 bp), heterozygous knockout mice (+/−, 200 bp and 600 bp) and homozygous knockout mice (−/−; 600 bp) are shown. (B) Weight gain in mice during PNU-282987 treatment. At the end of treatment, (C) HOMA-IR, (D) insulin tolerance test (ITT) and (E, F) glucose tolerance test (GTT) were performed. ITT was performed in mice 6 h after food removal with insulin challenge at 0.55 IU/kg of body weight (i.p.). GTT was performed in overnight fasted mice with glucose challenge at 2.0 g/kg of body weight (i.p.). Data are means ± SE (n = 5–6). ^#^ *P*<0.05, ^##^ *P*<0.01 *vs* saline treatment.

AMPKα2 and STAT3 have been reported to play important roles in regulating insulin sensitivity [37]–[41]. AMPKα2^−/−^ mice develop insulin resistance [42], [43]. To identify the involvement of AMPKα2 in the downstream signal pathway of α7-nAChR to enhance insulin sensitivity, we examined if activation of α7-nAChR in AMPKα2^−/−^ mice could improve insulin resistance. Fig. 6A illustrates the PCR products obtained with tail DNA from homozygous (−/−) and heterozygous (+/−) AMPKα2 knockout and wild-type (+/+) mice. The expected size of the AMPKα2^+/+^ amplicon is 200 bp, while that of the amplicon from AMPKα2^−/−^ mice is 600 bp.

As shown in Fig. 6, PNU-282987 treatment mildly reduced weight gain without statistical significance (Fig. 6B), but significantly reduced 60% HOMA-IR (Fig. 6C), increased glucose clearance in ITT and GTT (Fig. 6, D and E), while had no effect on insulin level in GTT (Fig. 6F). These results suggest that activation of α7-nAChR improves insulin sensitivity in AMPKα2^−/−^ mice, which rules out the involvement of AMPKα2. Moreover, these results combined with the results from normal rats (Fig. 1 and 2) and mice (Fig. 5) suggest that nicotine could modulate insulin sensitivity under both physiological and pathophysiological conditions.

**Figure 7:**
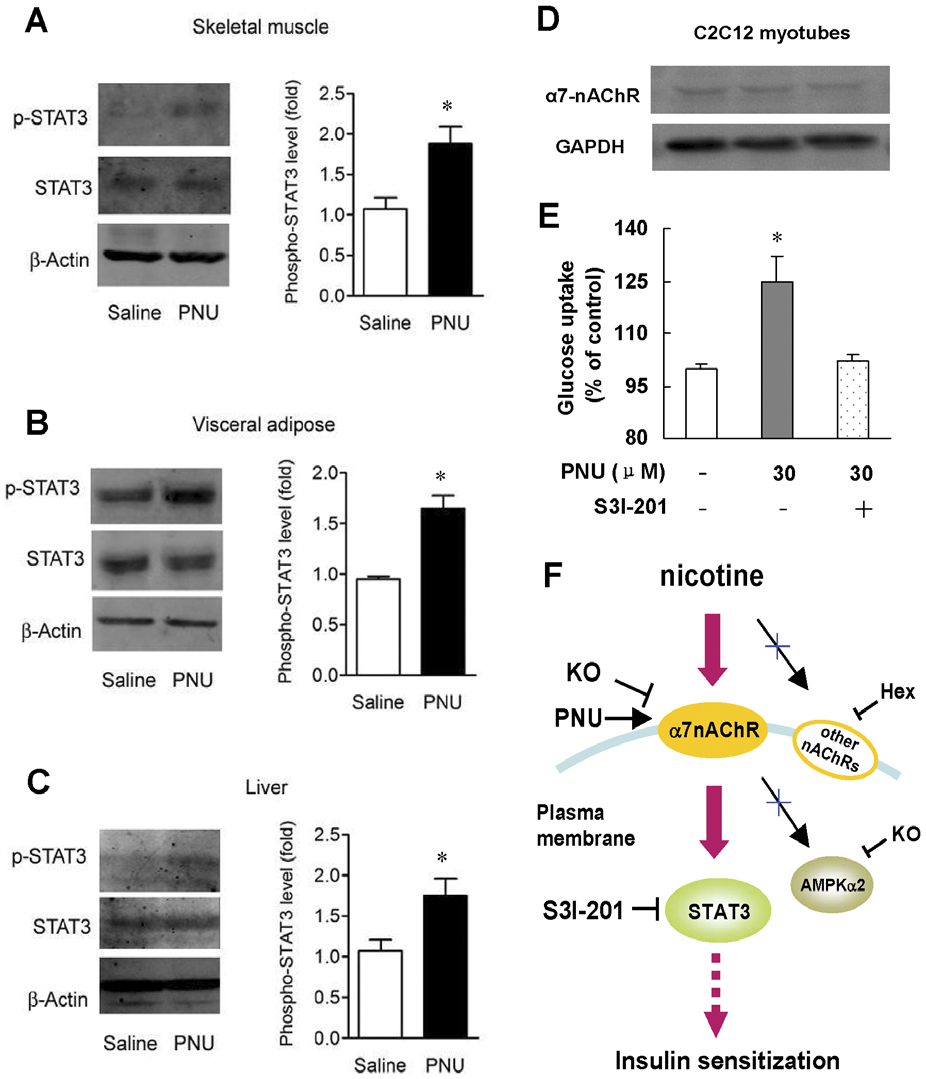
Selective α7-nAChR agonist, PNU-282987, enhances insulin sensitivity through activating STAT3. Western blots in (A) skeletal muscle, (B) visceral adipose and (C) liver in wild type mice after six weeks of saline or PNU-282987 treatment. Data are means ± SE (n = 5–6). ^*^ *P*<0.05 *vs* Saline. (D) Western blots of α7-nAChR protein in C2C12 myotubes. (E) PNU-282987 enhances glucose uptake in C2C12 myotubes, and this effect requires STAT3. Data are means ± SE (n = 4). ^*^ *P*<0.05 *vs* Control. (F) Proposed mechanism for nicotine-induced insulin sensitizing effect.

We then examined whether stimulating α7-nAChR lead to STAT3 activation. Western blotting showed that treatment of mice with PNU-282987 had no effect on STAT3 protein level but enhanced STAT3 phosphorylation in insulin target tissues, 1.78 fold, 1.72 fold, and 1.66 fold for skeletal muscle, adipose tissue and liver, respectively (Fig. 7A–C). To further verify the direct effect of α7-nAChR on insulin sensitivity and the involvement of STAT3, we performed test on C2C12 myotubes. Western blots verified the existence of α7-nAChR protein in C2C12 myotubes (Fig. 7D). As expected, insulin stimulated glucose uptake was enhanced 25% after PNU-282987 incubation. Moreover, the improved insulin stimulated glucose uptake by PNU-282987 was total impaired by specific STAT3 inhibitior, S3I-201 (Fig. 7E).

## Discussion

In the present study, we demonstrated that chronic treatment with nicotine enhanced insulin sensitivity in normal rats. We further showed that nicotine exerts insulin sensitizing effect through activating α7-nAChR-STAT3 signaling pathway. Activation of α7-nAChR not only improves insulin sensitivity in normal mice but also AMPKα2^−/−^ mice, an animal model of insulin resistance.

It has been reported that chronic nicotine administration can reduce insulin resistance in obese rats and mice [14], [44]. However, our current study demonstrated nicotine can improve insulin sensitivity in normal rats, which is a state different from obesity induced insulin resistance.

Previously, Swislocki et al showed that subcutaneous implanted nicotine pellets had no effect on insulin sensitivity in adult and juvenile rats [45], [46]. These discrepancies may be explained by a variety of factors, especially dosage, treatment duration, and route of nicotine administration [47]. The nicotine dose used in our study is 3 mg/kg/day roughly equal to 30% of theirs. Besides, the nicotine treatment time of ours is 3 weeks longer than theirs. Nicotine can activate multiple nAChR subtypes, and different nAChR subtypes share different dose sensitivity [48]. Nicotine was also reported to show different effect depending on different exposure time [15]. Thus, relatively low dose and long treatment time perhaps help to bring into play the insulin sensitizing effect of nicotine. As for nicotine delivery route, we used subcutaneous injection which is also different from implanted sustained release pellet in their studies. Thus, the above factors may explain the different outcome of nicotine on insulin sensitivity.

As majority of nicotine effects are mediated through nAChRs, we tried to identify specific nAChR subtype involved in insulin sensitizing effect of nicotine in this study. Our results from hexamethonium treatment ruled out the involvement of peripheral nAChRs, except for α7-nAChR, in the effect of nicotine. Further study with α7-nAChR^−/−^ mice provided evidence that the insulin sensitizing effect of nicotine was dependent on α7-nAChR. More importantly, the in vivo study using α7 selective nicotinic receptor agonist PNU-282987 soundly confirmed that activation of α7-nAChR could enhance insulin sensitivity.

We also noted that nicotine treatment can reduce body weight and blood triglyceride, but they did not seem to be involved in the insulin sensitizing mechanism of nicotine considering our animal models. Body weight reduction is associated with decreased food intake and increased energy expenditure. Recent findings demonstrate that nicotine decreases food intake and bodyweight through activating α3β4-nAChR [49]. Activation of STAT3 may mimic leptin signal in the brain to control food intake and energy expenditure [50]. Moreover, nicotine can enhance metabolic rate and activate uncoupling protein 1 in white and brown adipose tissue, a molecule which is important for adaptive thermogenesis and energy expenditure [51], [52]. However, in our experiment, deletion of α7-nAChR in mice abolished the insulin sensitizing effect of nicotine but not the bodyweight reducing effect of nicotine. α7-nAChR selective agonist improved insulin sensitivity not only in normal mice but also in insulin resistant AMPKα2^−/−^ mice without significantly reducing their bodyweight. Therefore, results from our study suggest that bodyweight loss is not a key contributor for nicotine induced insulin sensitization considering our animal models. Blood triglyceride reduction may also be the result of nicotine-induced increase in the metabolic rate and the fat tissue loss. Our previous study showed that fat tissue especially visceral fat tissue weight reduced a lot during nicotine treatment [16]. Besides, pretreatment of rats with hexamethonium reversed the triglyceride lowering effect of nicotine but had no effect on the insulin sensitizing effect, indicating that reduction of triglyceride may not involved in the insulin sensitizing effect of nicotine.

We further investigated α7-nAChR downstream molecule that may be involved in regulating insulin sensitivity. Results showed that STAT3 rather than AMPKα2 contributed to this pathway. We found that PNU-282987 treatment improved insulin sensitivity in AMPKα2^−/−^ mice, an animal model of insulin resistance. Although AMPKα2 plays critical role in controlling whole-body insulin sensitivity, and several insulin sensitizers, including thiazolidinediones and metformin, have been reported to activate AMPK [37], [38], our study provided evidence that it is not necessary for α7-nAChR activation to enhance insulin sensitivity. Moreover, it has been reported that α7-nAChR agonist, TC-7020, improved metabolic parameters in db/db mice, a model of obese type 2 diabetes. However, insulin sensitivity was not examined in that study [53]. Our current study demonstrated for the first time that α7-nAChR agonist had insulin sensitizing action not only in normal mice, but also in insulin resistant mice, i.e., AMPKα2^−/−^ mice, a model with no sign of inflammation and obesity, which is different from db/db mice [42].

STAT3 plays an important role in the regulation of insulin signaling pathway. It has been reported that inactivation of STAT3 contributes significantly to the pathogenesis of insulin resistance. STAT3 sensitizes the insulin signaling through suppression of glycogen synthase kinase-3β [39], a negative regulator of insulin signaling pathway. Our study showed that treatment of normal mice with α7-nAChR selective agonist PNU-282987 enhanced STAT3 phosphorylation in skeletal muscle, adipose tissue and liver. Moreover, the in vitro tests in C2C12 myotubes provide evidence that STAT3 is important for the insulin sensitizing effect of α7-nAChR. It should be noted that activating α7-nAChR has direct insulin sensitization effect in C2C12 myotubes. However, more evidence is needed to be provided on insulin target tissues. As GLUT4 plays an important role in insulin-stimulated glucose uptake [54], observing GLUT4 in C2C12 myotubes and the above animal models may contribute to understanding the mechanism of this effect.

Wang et al reported that nicotine ameliorates obesity-induced insulin resistance through suppressing inflammation in adipose tissue by activating cholinergic antiinflammatory pathway [44]. However, our results on non-obese animals did not associate with inflammation. Our gene chip assay showed that the mRNA expression of inflammatory cytokines in adipose tissue or muscle, such as TNFα, IL-6, IL-1β, iNOS and γ-IFN, are very low and shared no difference between nicotine treated and non-treated group (unpublished observations), and our previous studies showed that α7-nAChR^−/−^ mice express very low level of inflammatory cytokines [20]. No evidence indicates inflammation in AMPKα2^−/−^ mice, either [43].

Although the potential use of nicotine as therapeutic agent against insulin resistance is limited by its collateral toxicity, the application of α7-nAChR as a new target may be prospective. It will exert more specific effect while eluding collateral toxicity. Activating α7-nAChR may not only reduce obesity related insulin resistance by anti-inflammation mechanism [44], [53], but also ameliorate non-obese or low inflammation related insulin resistance through STAT3 related direct or indirect pathway. Besides, activating α7-nAChR has been demonstrated to benefit for treating many inflammation related diseases, such as arthritis, shock, stroke, myocardial infarction and Alzheimer's disease [17], [55]–[58], which will also contribute to the prevention and treatment of diabetic complications.

In conclusion, our study revealed that nicotine exerts its insulin sensitizing effect through α7-nAChR-STAT3 pathway which is independent of its anti-inflammatory effect (Fig. 7F). Developing new therapeutic method against insulin resistance based on α7-nAChR is worth further investigation in future.
